# Microglial activation is inhibited by selective anti-seizure medications

**DOI:** 10.1007/s00011-025-02076-7

**Published:** 2025-08-23

**Authors:** Robert Jürgen Platow, Sabrina Pommer, Julia Brauer, Yanyan Wang, Shyamala Mani, Angela M. Kaindl

**Affiliations:** 1 Institute of Cell Biology and Neurobiology, Charité—Universitätsmedizin Berlin, Berlin, Germany; 2 Department of Pediatric Neurology, Charité—Universitätsmedizin Berlin, Berlin, Germany; 3 Center for Chronically Sick Children, Charité—Universitätsmedizin Berlin, Berlin, Germany; 4 German Epilepsy Center for Children and Adolescents, Charité—Universitätsmedizin Berlin, Berlin, Germany; 5 Charité Pediatric Head and Neck Center, Charité—Universitätsmedizin Berlin, Augustenburger Platz 1, 13353 Berlin, Germany

**Keywords:** Epilepsy, Inflammation, Microglia, Anti-seizure medication

## Abstract

**Objective:**

To investigate the anti-inflammatory properties of anti-seizure medications (ASMs) administered to patients with drug-resistant epilepsy (DRE) and the role of sodium channels in microglial activation.

**Material:**

Primary microglia monocultures from mice brains.

**Treatment:**

Microglia were activated with 10 μg/mL lipopolysaccharide (LPS) or polyinosinic:polycytidylic acid (poly I:C) and pre- (45 min ASM then 2 h ASM plus stimulus) or post- (2 h stimulus then 24 h only ASM) treated with ASMs. Microglia were treated with cannabidiol (10 μM), stiripentol (250 μM), fenfluramine (50 μM), phenytoin (8 and 40 μM), cenobamate (300 and 900 μM), or the small molecule sodium channel blocker GS967 (10 and 30 μM). The sodium channel modulators tetrodotoxin (1 μM), µ-conotoxin KIIIA (1 μM), and β-pompilidotoxin (0.5 μM) were also applied.

**Methods:**

Microglia activation was quantified through measurements of *Ptgs2* (*Cox2*), *Tnf-α*, and *Ifn-β* induction by RT-qPCR and of cell morphology by immunocytochemistry. Expression of sodium channels in microglia was studied using PCR, RT-qPCR, immunohisto- and immunocytochemistry. Mann Whitney test and the Kruskal–Wallis test with Dunn’s multiple comparisons post-test were used.

**Results:**

ASMs have a differential effect on microglial activation. Uniquely, cenobamate inhibited the induction of *Ifn-β* and made the cells less amoeboid. The voltage gated sodium channel Na_v_1.2 is expressed by microglial cells and its expression levels change with microglial inflammatory response. Toxins that block sodium channels modulated microglial activation.

**Conclusions:**

ASMs, applied to patients with DRE, have a differential ability to reduce microglial activation and pro-inflammatory microglial morphology in vitro. Moreover, sodium channel blockage modulates inflammation through microglia activation. Taken together these results suggest, that further investigation of patient’s immune response to ASMs could be important.

**Supplementary Information:**

The online version contains supplementary material available at 10.1007/s00011-025-02076-7.

## Introduction

Epilepsy is one of the most common neurologic diseases world-wide [1]. Although there are now more than 30 anti-seizure medications (ASMs) available, around one third of epilepsy patients suffer from drug-resistant epilepsy (DRE), defined as two suitable ASMs not resulting in seizure freedom [2, 3]. The primary mechanism of most ASMs is through influencing neuronal excitability by increasing inhibitory and decreasing excitatory transmission [4]. However, considering the high number of patients with DRE, it is important to address additional mechanisms in epilepsy that could contribute to drug resistance.

One such mechanism is neuroinflammation that plays an important role in altering neuronal excitability and could be a factor contributing to DRE [5]. It is well established that seizure activity can result in inflammation, however recent evidence also points to inflammation playing a role in the process of epileptogenesis [6, 7]. In support of this, elevated levels of inflammatory cytokines have been observed in surgically resected epileptic foci from animal models and DRE patients, and neuroinflammation results in increased seizure susceptibility [5]. In line with the evidence that inflammation plays a role in DRE, steroids are often used to treat intractable epilepsies with positive outcomes in some cases [8–11].

Microglia are the resident immune cells of the brain that respond within minutes to acute neuronal hyperactivity in animal models of seizure induction [12]. These responses include morphological changes as well as release of pro-inflammatory cytokines that can directly affect neuronal excitability [13]. Significant microglial activation has been identified in resected brain tissue of children with intractable childhood epilepsy [14]. Microglial activation can also precede seizure onset, suggesting that they could play a causal role in disease pathogenesis [15]. In support of this microglia have been shown to affect neuronal excitability [16].

To investigate whether ASMs can differentially reduce inflammation, we chose to test ASMs, some of which have been associated with an anti-inflammatory effect and/or approved for treatment of Dravet syndrome (DS) and are often used to treat patients with DRE. These are cannabidiol, stiripentol, fenfluramine, phenytoin, cenobamate, and the experimental small molecule sodium channel blocker GS967. Cannabidiol, that has known anti-inflammatory properties, is approved for the treatment of the highly DREs Dravet syndrome (DS), Lennox Gastaut syndrome (LGS), and tuberous sclerosis complex (TSC) [17, 18]. Fenfluramine, approved for the treatment of LGS and DS, was shown recently to result in reduced microglial activation [19, 20]. Stiripentol is approved for treatment of DS and used as an add-on therapy in patients with drug-resistant focal epilepsy [21]. Phenytoin, applied in the treatment of status epilepticus and for long-term treatment in DRE, has been shown to reduce microglial activation [22, 23]. The new drug cenobamate is approved as adjunctive treatment in adults with focal-onset epilepsy and we have further shown an effect in individuals with DS [24]. GS967 was shown to be highly effective in reducing seizure frequency in a mouse model of DS [25].

Taken together, we hypothesized that these ASMs may reduce inflammation, resulting in a lowered threshold for seizures. Here, we test this hypothesis by studying the effect of these ASMs directly on microglial activation in vitro. This is important because this points to reduction of microglial activation as a new target for the treatment of DRE and may influence the choice of a specific ASM.

## Material and methods

### Isolation and culture of primary microglia

Mice pups were acquired from the Charité Experimental Medicine Research Facility (FEM). P0-P4 pups of both sexes from either *C57BL/6 J* or *C57BL/6N* strain were used. The protocol modified from the reference is given in the supplementary information [26]. 12 days after seeding of mixed cultures in in Dulbecco's Modified Eagle Medium (DMEM) with 10% fetal bovine serum (FBS) and 1% penicillin–streptomycin (Pen/Strep) (DMEM-C), microglia were separated from astrocytes by shaking cell culture flasks for 25 min at 250 rpm. The supernatant was collected, cells pelleted and resuspended in DMEM-C. Cells were counted and seeded into 24 well plates at the required number (for RT-qPCR: 150.000 cells per well of a 24-well plate, for immunocytochemistry 30.000 cells per coverslip, coated with poly-D-lysine (PDL)). After 24 h, the medium was replaced with Macrophage-SFM (serum free medium) containing 1% Pen/Strep, and the cells were stimulated 2–4 days after seeding. The microglia monoculture was stained for other cell types to assess purity. Glial fibrillary acidic protein (GFAP) was used for astrocytes, myelin basic protein (MBP) and neural/glial antigen 2 (NG2) for oligodendrocyte cell lineage, and class III β-tubulin (TUJ1) for neuronal presence. By these criteria we had a microglia purity of over 98% (see supplementary Fig. 1).

### Stimulation of microglial cultures

To investigate the effects of the drugs on inflammatory processes, we triggered inflammation by stimulating the cells with lipopolysaccharide (LPS (*E. coli* serotype O111:B4)) at 10 μg/mL, as a model for bacterial infection, or with polyinosinic: polycytidylic acid (poly I:C) at 10 μg/mL, as a model of viral infection. Two paradigms were used: (i) prophylactic paradigm: In the first paradigm, microglia were exposed to either the drug or vehicle control for 45 min before adding the stimulus. Cells were incubated for 6 h, after which medium was removed or collected and stored at − 70 ℃ for cytokine assay. The plate with the cells was stored at − 70 ℃ until analyzed for prostaglandin-endoperoxide synthase 2 (*Ptgs2 or Cox2*) and tumor necrosis factor-α (*Tnf-α*) or interferon-β (*Ifn-β*) levels. (ii) Therapeutic paradigm: In the second paradigm, microglia were stimulated for 2 h, after which the wells were rinsed once in Macrophage-SFM and then incubated with either drug or vehicle control for 24 h, after which the plates were processed for cytokines as mentioned previously (graphic of experimental paradigm is provided in supplementary information). Cells on coverslips, for immunocytochemistry, were fixed with 4% paraformaldehyde (PFA) in phosphate buffered saline (PBS) (15 min at room temperature), washed three times with cold PBS and then stored in PBS at 4 ℃, until staining. The drug concentrations finally tested are the result of preliminary experiments in which a range of concentrations were tested for effect. Stock concentrations, solvent information, and preliminary concentration range tested can be found in the supplementary tables.

### RNA extraction, cDNA synthesis and quantitative real-time PCR

The NucleoSpin RNA XS Kit (Macherey–Nagel, Düren, Germany) was used for RNA extraction and the RNA was reverse transcribed into cDNA, using the cDNA Synthesis Kit (Bio-Rad, Hercules, USA), all according to manufacturer’s protocol. For RT-qPCR, the iTaq™ Universal SYBR® Green Supermix (Bio-Rad) with specific primers were used. Primer sequences and references for them are given in supplementary tables [27, 28]. *Ptgs2* and *Tnf-α* levels were determined after LPS stimulation and *Ifn-β* after poly I:C stimulation. *Rpl13a* expression was determined as a reference gene for normalisation of mRNA expression.

### Detection of Scn isoforms

1 µL of cDNA was used as template in a 20 µL PCR reaction. PCR products along with 100 bp ladder (Thermo fisher) were run on the 2% agarose gel, prepared in 1 × TBE (Tris/Borate/EDTA) buffer. Primer sequences used are given in supplementary tables.

### Immunocytochemistry

Briefly after fixation cells were permeabilised for 15 min in 0.1% Triton + 3% bovine serum albumin (BSA) in PBS, blocked for one hour with 10% donkey serum and 3% BSA in PBS. The cells were incubated for 2 h at room temperature (RT) with primary antibody in 1:2 diluted blocking solution. The cells were washed 3 times with PBS for 10 min each, then incubated for 1.5 h at RT, in 1:2 diluted blocking solution, with secondary antibody. The cells were washed 3 × for 10 min each with PBS and then incubated for 15 min at RT with DAPI, washed twice for 10 min with PBS and briefly rinsed with water and mounted in Fluoromount. Antibodies used and their dilutions are specified in the supplementary tables.

### Immunohistochemistry

Brains were removed from P14 and P56 mice and fixed in 4% PFA at 4 ℃ overnight and transferred to 30% sucrose solution. O.C.T. (Optimal cutting temperature compound) was used to embed brains. 12 µm brain slices were sectioned and blocked with 10% donkey serum/0.1% Triton X-100 in PBS at RT for 1 h in a humid box. Sections were incubated with primary antibodies at 4 ℃ overnight, washed 3 × for 10 min with PBS and incubated with secondary antibody at RT for 2 h. The slides were washed three times with PBS and mounted with Fluoromount. Antibodies used and their dilutions are specified in the supplementary tables.

### Morphological analysis

Pictures were taken using an Olympus BX50 microscope, coupled with an HBO® Mercury short arc lamp (Osram HBO® 103 W/2) and a MagnaFire camera with MagnaFire software 2.1. Analysis was done using Fiji (ImageJ) version 2.16.0.

### Analysis by cytokine assay

Release of selected cytokines and chemokines into the medium was analysed using the Proteome Profiler Mouse Cytokine Array Kit, Panel A from R&D Systems™ (Minneapolis, MN, USA). Prior to analysis, the medium was centrifuged at 1000 rcf for 5 min at 4 ℃. The analysis was performed according to the manufacturer's protocol. Imaging was done using Azure 600 Imaging System (Biozym®, Hessisch Oldendorf, Germany) and the analysis was done using the QuickSpots program version 25.6.0.3.

### Graphics

Graphics were built using GraphPad Prism version 10.2.3 and Adobe Illustrator version 29.4.

#### Statistics

The RT-qPCR and morphology experiments were performed with a minimum of three biological replicates with duplicate or triplicate wells for each biological replicate. For statistics each biological replicate was counted as a separate n. RT-qPCR was performed in duplicate from each well and *Rpl13a* was used as control for quantification normalization. Analyses were performed with the StepOne software V2.3 (Thermo Fisher Scientific, Waltham, MA, USA) and a relative quantification approach was used, according to the 2-ddCT method [29]. For the histograms the average 2-ddCT value for each well was plotted. For morphological analysis at least 5–10 random fields per coverslip were analysed for a total of > 100 cells per coverslip. This was done for three biological replicates. The average area and circularity of the cells in each field was measured and plotted. When comparing across two groups the non-parametric Mann Whitney test was used. When comparing across more than two groups the Kruskal–Wallis test was used with Dunn's multiple comparisons post-test. P values are reported and the histograms represent mean ± standard error of the mean (S.E.M). Biological triplicates (n = 3) were used for the analysis of the cytokine assay. The mean value of the measured pixel density in the medication groups was compared with that of the respective vehicle control group. The pixel density of the vehicle control was normalized as 1 and the values of the medication were normalized accordingly. For statistical analysis, an unpaired t-test with Holm-Šídák correction was used to adjust for multiple testing.

## Results

### Effect of ASMs on inflammation markers in microglia

**Fig. 1:**
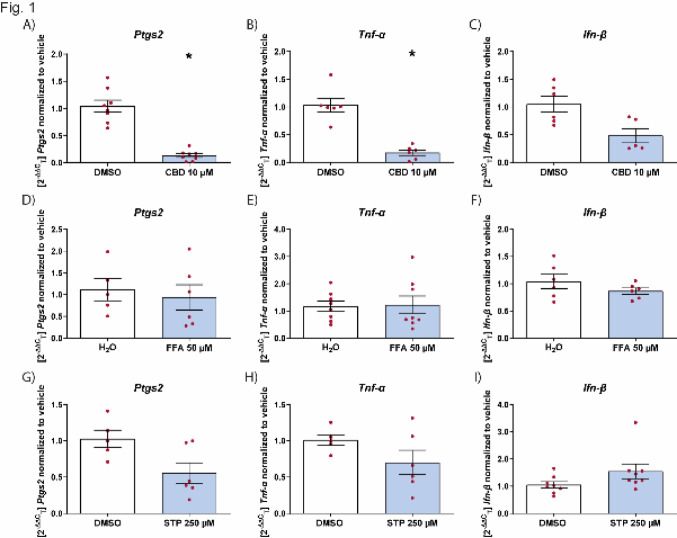
Effect of ASMs on inflammation markers in microglia. Effect of CBD (**A**-**C)**, FFA (**D**-**F)** and STP (**G**-**I)** on the mRNA levels of *Ptgs2*, *Tnf-α* or *Ifn-β* on microglial cells stimulated with LPS or poly I:C. Levels of *Ptgs2* (**A**, **D** and **G**), *Tnf-α* (**B**, **E** and **H**) and *Ifn-β* (**C**, **F** and **I**) normalized to vehicle control in microglia pretreated with the drug for 45 min, followed by LPS (10 μg/mL) (**A**, **B**, **D**, **E**, **G** and **H**) or poly I:C (10 μg/mL) (**C**, **F** and **I**) for 6 h. Data are expressed as mean ± SEM (n = 3) *p < 0.05. *CBD* cannabidiol, *DMSO* dimethyl sulfoxide, *FFA* fenfluramine, *STP* stiripentol

As a positive reference for the suitability of our assay, we first tested whether cannabidiol could reduce microglial activation induced by LPS [17]. As a readout, the effect of the drug was assessed on *Ptgs2* and *Tnf-α* levels, whose levels have been studied in the context of epilepsy [30]. Cannabidiol reduced the level of *Ptgs2* and *Tnf-α* when given before LPS stimulation of microglia (Fig. 1A and B). We also used poly I:C, that induces the interferon pathway, as a second activation stimulus. Using the levels of *Ifn-β* as a readout, cannabidiol had no effect on reducing *Ifn-β* levels (Fig. 1C).

We then examined the effect of fenfluramine, that has been shown in vivo to reduce the amount of activated microglia [20]. We initially tested concentrations ranging from 0.1–100 µM and found that the variability was the least at 50 µM. Therefore, this concentration was used for further testing. Fenfluramine was not effective in suppressing inflammation when applied prophylactically directly on microglia (Fig. 1D–F), suggesting that reduction in microglia activation seen in vivo, was potentially a consequence of seizure reduction [20]. For stiripentol, we tested concentrations between 10 and 250 µM and used 250 µM, based on initial results. We showed that, as with fenfluramine, stiripentol also does not have any effect on microglial activation (Fig. 1G–I).

### Effect of sodium channel blockers on inflammation markers in microglia

**Fig. 2:**
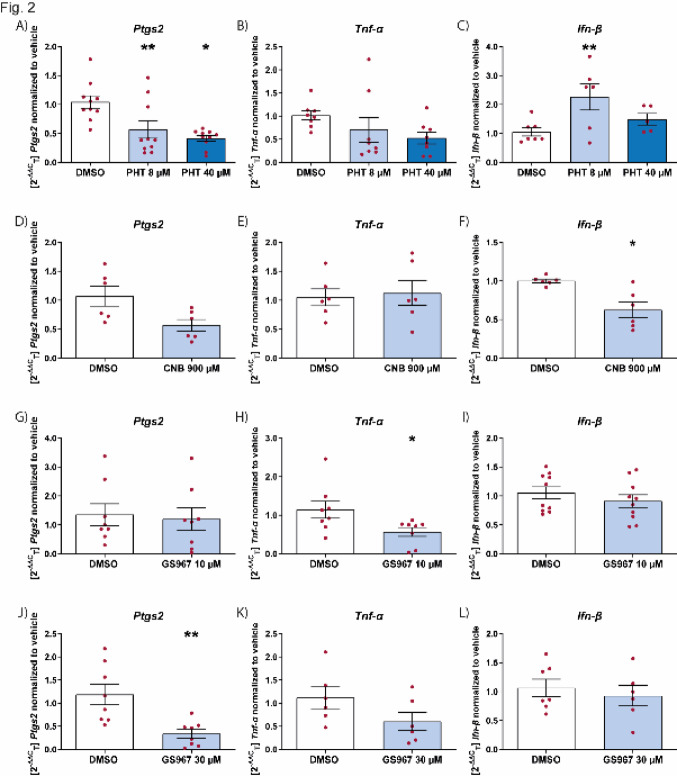
Effect of sodium channel blockers on inflammation markers in microglia. Effect of PHT (**A**-**C)**, CNB (**D**-**F)** and GS967 (**G**-**L)** on the mRNA levels for *Ptgs2*, *Tnf-α* or *Ifn-β* on microglial cells stimulated with LPS or poly I:C. Levels of *Ptgs2* (**A**, **D**, **G**, **J**) and *Tnf-α* (**B**, **E**, **H**, **K**) and *Ifn-β* (**C**, **F**, **I**, **L**) normalized to vehicle control in microglia pretreated with drug for 45 min, followed by LPS (10 μg/mL) (**A**, **B**, **D**, **E**, **G**, **H**, **J**, **K**) or poly I:C (10 μg/mL) (**C**, **F**, **I**, **L**) for 6 h. Data are expressed as mean ± SEM (n = 3) *p < 0.05; **p < 0.01. *CNB* cenobamate, *DMSO* dimethyl sulfoxide, *PHT* phenytoin

We examined the effect of the sodium channel blockers phenytoin, cenobamate, and GS967, that have been shown to reduce seizure frequency in animal models and patients with the DRE Dravet syndrome [24, 25, 31]. Phenytoin was able to suppress the induction of *Ptgs2* but not *Tnf-α* following LPS stimulation (Fig. 2A and B), and it had a significant effect on the upregulation of *Ifn-β,* when microglia were stimulated with poly I:C (Fig. 2C). Cenobamate did not have a significant effect on *Ptgs2* or *Tnf-α* levels when stimulated with LPS (Fig. 2D and E), but had a significant effect on reducing *Ifn-β* when microglia were stimulated with poly I:C (Fig. 2F). Thus, unlike cannabidiol or phenytoin, cenobamate had a reducing effect on the innate immune interferon response of microglia, as shown by the decrease in *Ifn-β* (Fig. 2F). In contrast to cenobamate but similar to cannabidiol, GS967 was able to suppress the pro-inflammatory effect of LPS stimulated microglia (Fig. 2G and H) but not to microglia stimulated with poly I:C (Fig. 2I). Taken together, sodium channel blockers also affect the interferon pathway (Fig. 2C and F) unlike the anti-inflammatory effect of cannabidiol (Fig. 1C).

### Effect of ASMs on cyto- and chemokines in microglia

**Fig. 3:**
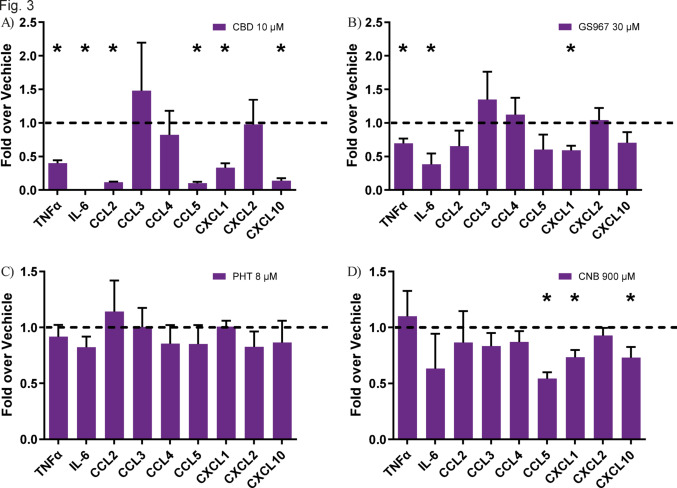
Effect of sodium channel blockers on cyto- and chemokines in microglia. Effect of CBD (**A**), GS967 (**B**), PHT (**C**) and CNB (**D**) on the release of selected cyto- and chemokines on microglial cells, pretreated with the drug for 45 min, followed by LPS (10 μg/mL) (**A**, **B** and **C**) or poly I:C (10 μg/mL) (**D**) for 6 h. Cyto- and chemokine levels are expressed relative to their respective vehicle controls, which were normalized to a value of 1. (n = 3) (Exemplary pictures of the membranes can be found in supplementary Fig. 2). *CBD* cannabidiol, *CNB* cenobamate, *PHT* phenytoin

To validate the RT-qPCR readout, we used the mouse cytokine array to measure the amount of cytokines secreted into the medium. For that, we used the medium from wells that showed a significant inflammatory reduction in the prophylactic paradigm. We found that, in line with the RT-qPCR result, cannabidiol results in a greater reduction in TNF-α levels than GS967 and this trend was also seen for IL-6 (Fig. 3A and B). Cannabidiol further had an effect on various chemokines (Fig. 3A). We then performed the cytokine array for cenobamate, stimulating the cells with poly I:C. We showed that like GS967, cenobamate reduced the level of the chemokines CXCL1 but also reduced the levels of CCL5 and CXCL10 like cannabidiol (Fig. 3D). Interestingly, the sodium channel blocker phenytoin had no effect.

### Effect of ASMs on microglial activation in a therapeutic paradigm

**Fig. 4:**
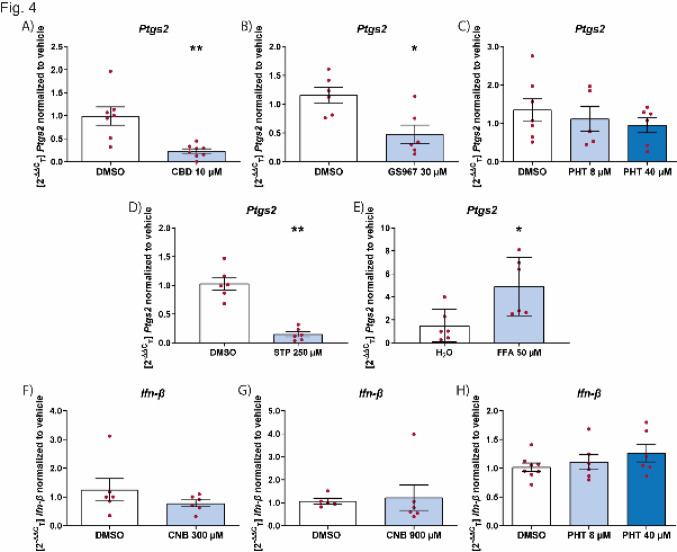
Effect of ASMs on inflammation markers on preactivated microglia. Effect of CBD (**A**), GS967 (**B**), PHT (**C** and **H**), STP (**D**), FFA (**E**) and CNB (**F** and **G**) on the mRNA levels of *Ptgs2* (**A**-**E**) on microglial cells pre-stimulated with LPS (10 μg/mL) for 2 h and *Ifn-β* mRNA levels (**F**–**H**) after pretreatment with poly I:C (10 μg/mL) for 2 h. RT-qPCR analysis was performed 24 h after change to only drug. Data are expressed as mean ± SEM (n = 3) *p < 0.05; **p < 0.01. *CBD* cannabidiol, *CNB* cenobamate, *DMSO* dimethyl sulfoxide, *FFA* fenfluramine, *PHT* phenytoin, *STP* stiripentol

We next tested the ability of the ASMs to reduce inflammation in already activated microglia (therapeutic paradigm). We showed that cannabidiol was also able to effectively suppress inflammation when given after the onset of inflammation (Fig. 4A). This was also the case for GS967 (Fig. 4B) but not for phenytoin (Fig. 4C). Also, when prestimulated with poly I:C neither cenobamate nor phenytoin had any effect on *Ifn-β* levels (Fig. 4F–H). Intriguingly, in contrast to the prophylactic administration, stiripentol showed a significant anti-inflammatory effect (Fig. 4D), while fenfluramine led to an increase in *Ptgs2* levels (Fig. 4E).

### Effect of anti-seizure medication on microglial morphology

**Fig. 5:**
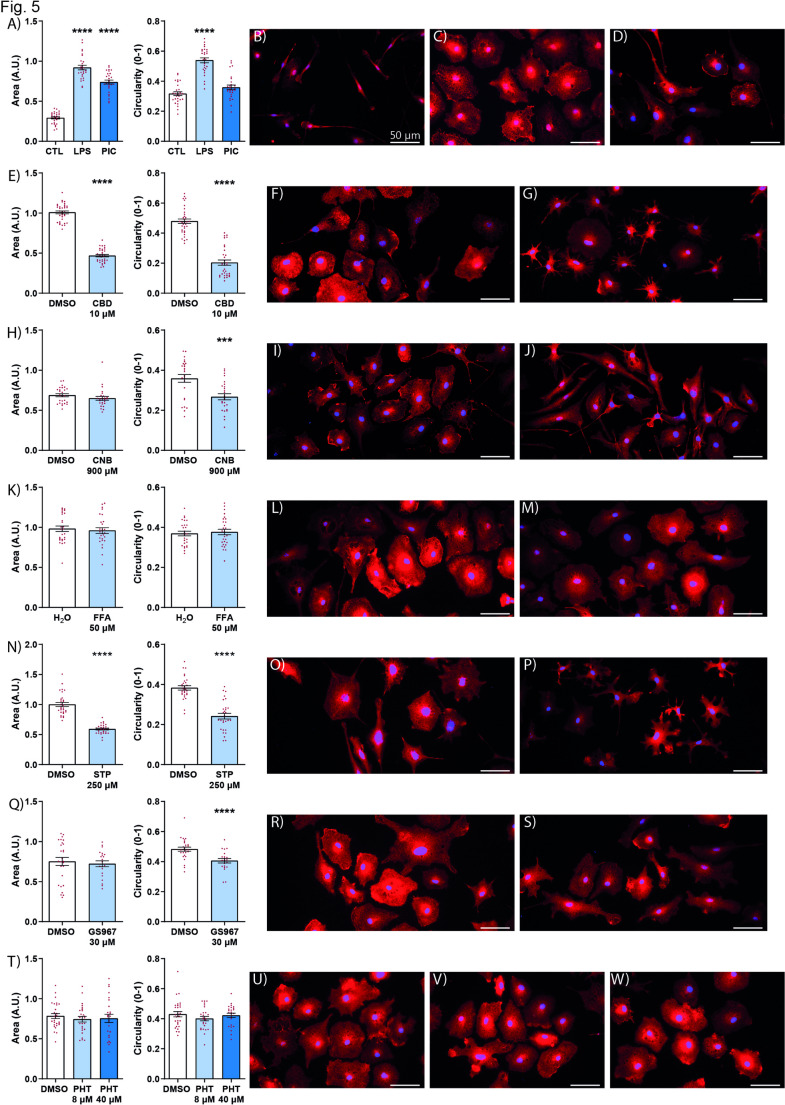
Effect of ASMs on microglia morphology. Quantitation of area (**A** left) and circularity (**A** right) and representative field for CTL (**B**) LPS (**C**) poly I:C (**D**) after cells were stimulated with either LPS or poly I:C. Quantiation of area (**E** left) and circularity (**E** right) and representative field for vehicle (**F**) and CBD (**G**). Quantiation of area (**H** left) and circularity (**H** right) and representative field for vehicle (**I**) and CNB (**J**). Quantiation of area (**K** left) and circularity (**K** right) and representative field for vehicle (**L**) and FFA (**M**). Quantiation of area (**N** left) and circularity (**N** right) and representative field for vehicle (**O**) and STP (**P**). Quantiation of area (**Q** left) and circularity (**Q** right) and representative field for vehicle (**R**), GS967 (**S**). Quantiation of area (**T** left) and circularity (**T** right) and representative field for vehicle (**U**), 8 µM PHT (**V**) and 40 µM PHT (**W**). Treatment initiated 2 h after microglial activation. All except for CNB were activated with LPS 10 µg/mL. For CNB activation was by poly I:C 10 µg/mL. Cells were fixiated with PFA 24 h after treatment. Immunocytochemistry for IBA1 (red), nuclei DAPI (blue). Merged images shown. Graphs represent mean ± S.E.M. ***p < 0.001; ****p < 0.0001. *CBD* cannabidiol, *CNB* cenobamate, *CTL* control, *DMSO* dimethyl sulfoxide, *FFA* fenfluramine, *LPS* lipopolysaccharide, *PHT* Phenytoin, *PIC* poly I: *C* polyinosinic:polycytidylic acid, *STP* stiripentol, *IBA1* ionized calcium-binding adapter molecule 1

Microglial morphology is dynamic and can reflect functional changes, such that a larger cell body and more circular morphology indicates an amoeboid state, that performs a phagocytoc function [32]. Using the therapeutic paradigm, in which microglia were treated with ASMs after the onset of inflammation, we quantified the changes in microglial morphology due to the drug. We first showed that cannabidiol had a significant effect on reducing both cell area and circularity (Fig. 5E–G) making them closer to the resting state [33]. The difference we found between fenfluramine and stiripentol, in their ability to reduce existing inflammation (Fig. 4D and E), was also evident in their effect on the morphology of microglia (Fig. 5K–P). While stiripentol had a significant effect on microglial morphology (Fig. 5K–M), fenfluramine had no effect (Fig. 5N–P). In terms of sodium channel blockers, both cenobamate and GS967 reduced the circularity, a measure of the amoeboid shape of microglia (Fig. 5H–J, Q–S). Phenytoin, however, did not have any effect on microglia morphology (Fig. 5T–W).

### Effect of anti-seizure medication on cytokine profile

**Fig. 6:**
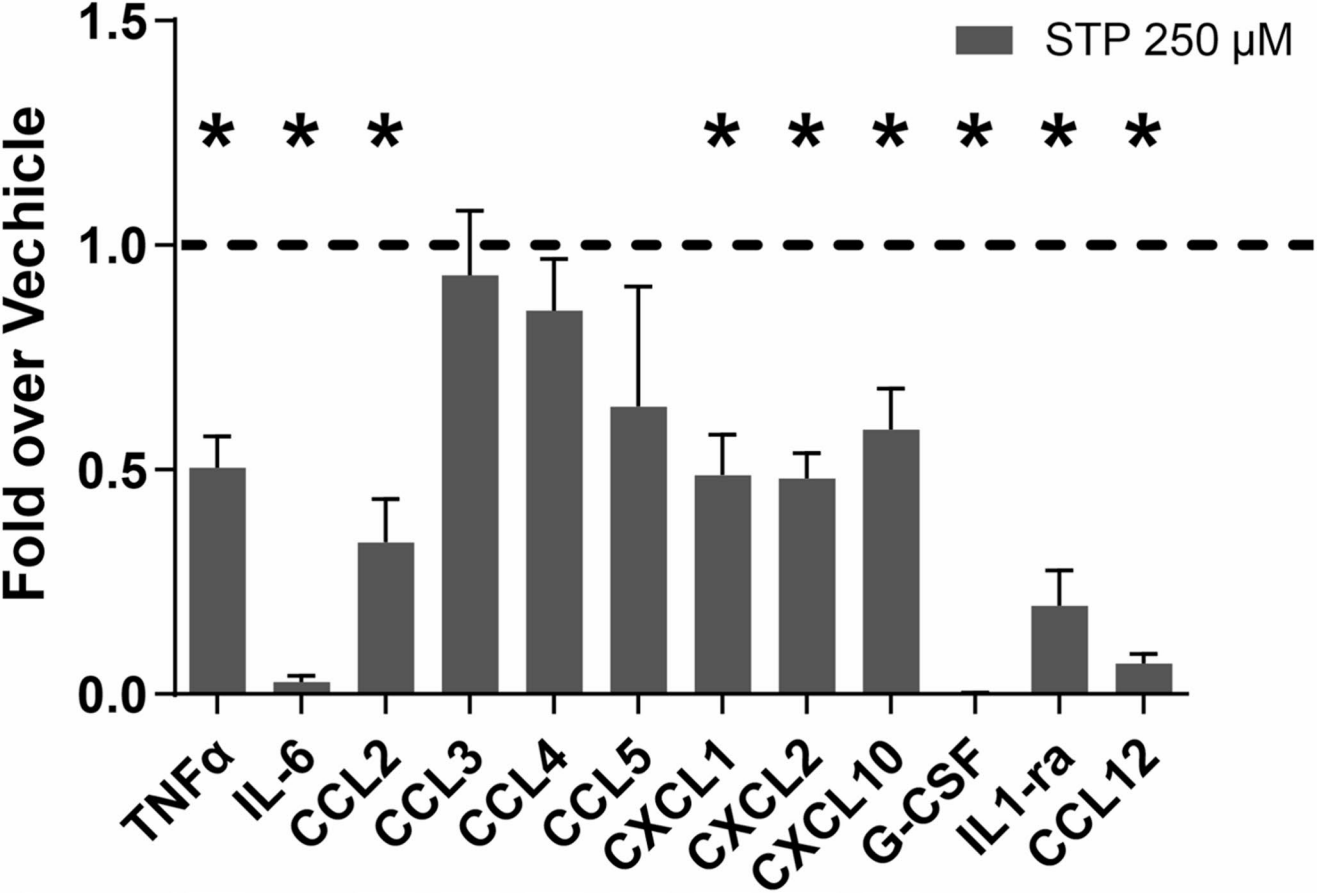
Effect of stiripentol on cyto- and chemokines in microglia. Effect of STP on the release of selected cyto- and chemokines on microglial cells pre-stimulated with LPS (10 μg/mL) for 2 h followed with only STP for 24 h. Cyto- and chemokine levels are expressed relative to their respective vehicle controles, which were normalized to a value of 1. (n = 3) *STP* stiripentol

Having seen the effect of stiripentol on microglial morphology (Fig. 5N–P) and suppression of inflammation (Fig. 4D), we looked at whether this was reflected in changes in the cytokine profile at this later time point. We showed that stiripentol had an effect on reducing several pro-inflammatory cytokines and chemokines (Fig. 6).

### Effect of voltage-gated sodium channel modulators on microglial activation

**Fig. 7:**
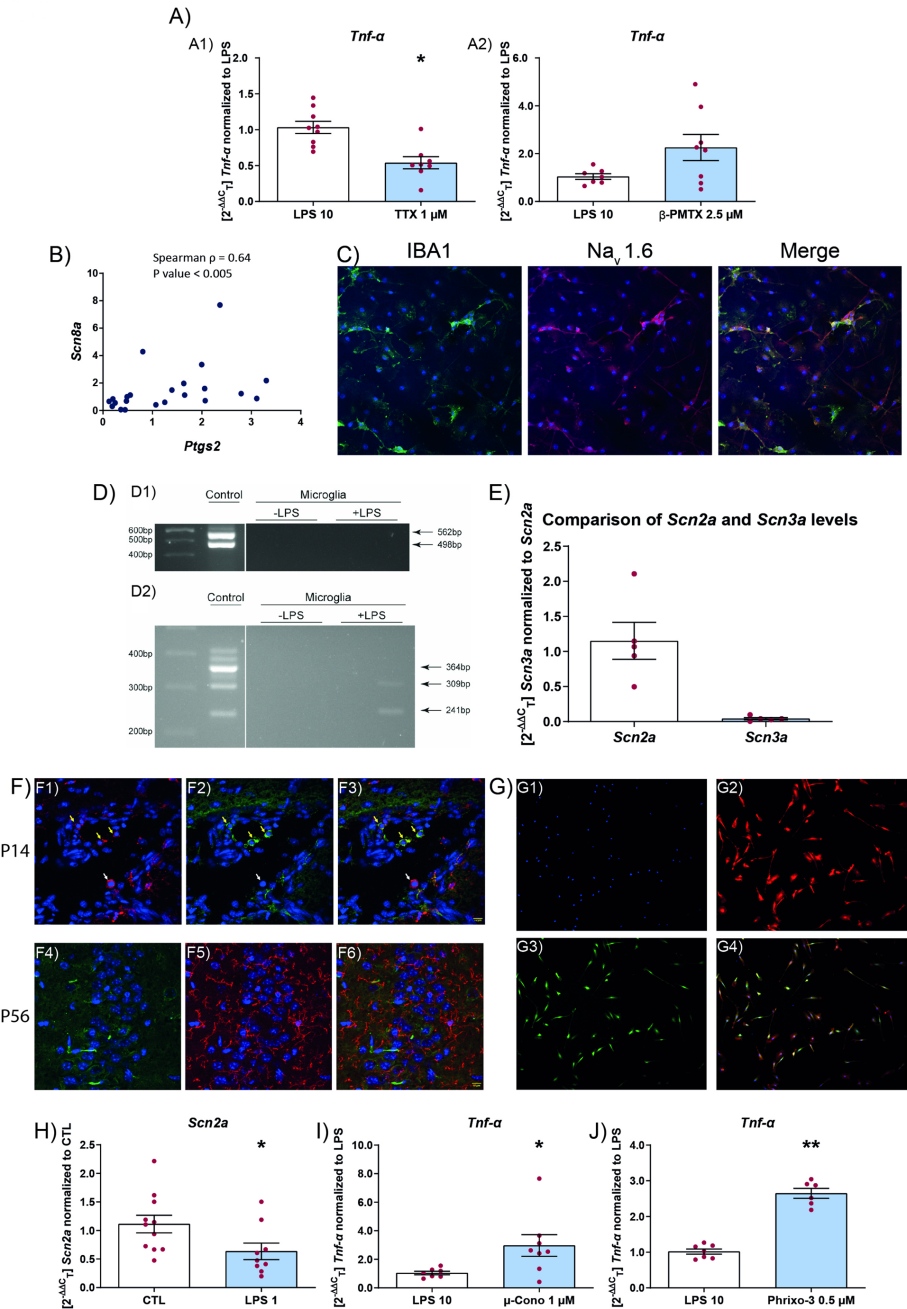
Sodium channel modulator effect on microglia. **A** Effect of TTX (**A1**) and β-PMTX (**A2**) on the mRNA levels of *Tnf-α* on microglial cells stimulated with LPS. Levels of *Tnf-α* normalized to LPS treated microglia, microglia were pretreated with toxin for 45 min, followed by LPS (10 µg/mL) for 6 h. Data are expressed as mean ± SEM (n = 3) *p < 0.05. **B** Spearman Rho between *Scn8a* and *Ptgs2* mRNA levels (**B**). **C** expression of Na_v_1.6 in activated microglia, LPS (1 µg/mL) treated for 24 h (**C**). Immunocytochemistry for IBA1 (green), Na_v_1.6 (red), nuclei DAPI (blue). **D** Brain (control) and microglia untreated (-LPS) or treated (+ LPS (1 µg/mL)) for 24 h. 564 bp = *Scn1a* transcript with poison exon and 498 bp without inclusion of the poison exon (**D1**). Brain (control) and microglia untreated (-LPS) or treated (+ LPS (1 µg/ml)) for 24 h. 364 bp = *Scn8a* full length transcript, 309 bp = *Scn8a* transcript with poison exon, 241 bp = *Scn8a* transcript that skips exon (**D2**). **E** Comparison of *Scn2a* and *Scn3a* mRNA levels in microglia (**E**). **F** Expression of Na_v_1.2 in mouse in vivo in microglia (**F**), P14 (**F1**–**F3**), P56 (**F4**–**F6**). **G** Expression of Na_v_1.2 in vitro in microglia (**G**). **F** and **G** Immunohisto- and immunocytochemistry for IBA1 (red), Na_v_1.2 (green), nuclei DAPI (blue). **H** *Scn2a* mRNA levels after 24 h of activation by LPS (1 µg/mL) (**H**). **I** and **J** Microglia pretreated with either µ-Cono (**I**) or Phrixo-3 (**J**) for 45 min followed by LPS (10 μg/mL) for 6 h. Data are expressed as mean ± SEM (n = 3) *p < 0.05; **p < 0.01. *CTL* control, *LPS* lipopolysaccharide, *µ-Cono* µ-conotoxin KIIIA, *Phrixo-3* phrixotoxin-3, *TTX* tetrodotoxin, *β-PMTX* β-pompilidotoxin, *IBA1* ionized calcium-binding adapter molecule 1

Mutations in sodium channels result in some of the most severe forms of epilesy and almost always they tend to be drug resistant with inflammation playing a critical role [34]. Therefore we wanted to test the role of sodium channels in microglia activation. Previously it was shown that tetrodotoxin could modulate the activity of voltage-gated sodium channels (VGSCs) on microglia [35, 36]. Since we saw an effect of sodium channel blockers on microglial activation, we tested whether tetrodotoxin was also able to modulate activation under similar conditions. In addition, we also used β-pompilidotoxin to enhance the activity of VGSCs [37]. In accordance with previous results, tetrodotoxin was able to reduce *Tnf-α* levels (Fig. 7A1). However, application of β-pompilidotoxin resulted in no increase in *Tnf-α* levels over LPS alone (Fig. 7A2).

As to the identity of the VGSCs, previous work has implicated the role of Na_v_1.6 in microglial activation [22, 35, 38]. Therefore, we tested whether the reduction in the mRNA levels of *Ptgs2* was correlated with a reduction in *Scn8a* mRNA. For this, we took the drug conditions of GS967 at 10 and 30 µM and CNB 900 µM at 6 and 24 h and show significant Spearman’s correlation coefficient between levels of *Ptgs2* and *Scn8a* (Fig. 7B). Since Na_v_1.6 was detected at low levels in control conditions, we confirmed Na_v_1.6 expression in microglial culture in conditions where they were stimulated with LPS (1 µg/ml) for 24 h (Fig. 7C).

Next we used primers to differentiate between the *Scn8a* isoforms to study which isoform is expressed in microglial cells. We found that microglia did not express the full-length mRNA that codes for the full-length protein, that forms the functional channel. However the delta 18 and the poison exon transcript of *Scn8a* could be detected (Fig. 7D2) [39, 40]. We did not detect *Scn1a* in microglia using primers that detected all isoforms of the *Scn1a* mRNA (Fig. 7D1) [41]. 

To test whether other sodium channels, that are linked to DRE, are present on microglia, we performed RT-qPCR for *Scn2a* and *Scn3a*. We detected *Scn2a* transcripts by RT-qPCR (Fig. 7E) and expression by immunohistochemistry (Fig. 7F). We also detected *Scn3a* mRNA at very low levels compared to *Scn2a* (Fig. 7E). Further we show, by immunocytochemistry, that microglia in culture express Na_v_1.2 (Fig. 7G). In vivo, we found that a subset of microglia express Na_v_1.2 at P14 but not in mature adult microglia at P56 (Fig. 7F). We showed that Na_v_1.2 may modulate inflammation in microglia by showing that inflammation reduces *Scn2a* mRNA (Fig. 7H) and conversely blocking Na_v_1.2 results in increased inflammation (Fig. 7I and J). For this, we used two different Na_v_1.2 blockers µ-conotoxin KIIIA and phrixotoxin-3 [37, 42] that have previously been used at these concentrations to block Na_v_1.2 activity.

## Discussion

Management of DRE is an unsolved clinical problem. This is especially pertinent when it comes to childhood epilepsy, since repeated seizures can result in developmental slowing and/or regression. There has been a lot of interest recently on the role of neuroinflammation in DRE. One compound known to have anti-inflammatory properties, cannabidiol, is an ASM approved for the treatment of DRE subtypes. Therefore, in this paper we address the question of whether other ASMs, that have shown benefit in patients with DRE, also exhibit anti-inflammatory properties, as this may stimulate investigation into patient's immune response to ASMs.

To study the direct effect of ASMs on neuroinflammation and not reduced inflammation as a consequence of reduced seizure activity, we exposed pure primary microglia in culture to different ASMs. The concentrations used in our experiment was chosen based on preliminary testing. We used, as our primary readout, the well-established PTGS2 pathway, that is involved in both generation of seizures and progression of epilepsy [43]. We used the endotoxin LPS, which mimics a bacterial infection, as a stimulus. LPS is the ligand for the TLR4 (Toll-like receptor 4) that promotes nuclear factor'kappa-light-chain-enhancer' of activated B-cells (NF-κb) signalling and production of PTGS2 and TNF-α. We also used a dsRNA viral mimic that binds to TLR3 and stimulates the production of type I interferon by a MyD88 (Myeloid differentiation primary response 88) independent, IRFs (interferon regulatory factors) dependent pathway. These two pathways can be differentially modulated by phytocannabinoids [44]. 

We first showed that cannabidiol, at concentrations previously shown to reduce inflammation, can indeed directly act on microglia, and has an anti-inflammatory effect, whether administered prophylactically or after activation of microglia [17]. However, cannabidiol had no effect on the induction of *Ifn-β*. The sodium channel blockers GS967 and cenobamate both had an effect on microglial inflammation, although their effects were different. While GS967 reduced the levels of *Tnf-α* and IL-6 cenobamate had an effect on *Ifn-β* and CXCL10. Both sodium channel blockers reduced the levels of CXCL1 which may be of significance since the CXCL1-CXCR1/2 signaling has been shown to play a role in seizures [45]. Interestingly, phenytoin had no effect. Of the substances we tested, the ASM cenobamate, at a concentration previously shown to inhibit sodium currents, was the only one that significantly reduced *Ifn-β*, an important component of the innate immune response of microglia [46]. This pathway can also be activated by damage to cells caused by seizures and could therefore constitute a target that may be important to address [27, 47]. 

Cytokine assays showed that cenobamate behaved similarly to cannabidiol by reducing the levels of Ccl5 and Cxcl10, both of which have been known to be mediators of inflammation in epilepsy [48]. One of our most striking findings is the anti-inflammatory effect of stiripentol, that has not been reported so far. In addition, we also analyzed the morphology of microglia since a larger cell area, that is more circular, indicates an amoeboid morphology that is phagocytotic [32]. Intriguingly, we found that both cenobamate and GS967 reduced circularity. Stiripentol and fenfluramine, however, had different effects on microglial morphology, which was also observed in their differential effect on microglial activation. 

Since our results showed that sodium channel blockers could modulate inflammation and previous work had implicated the presence of Na_v_1.6 in microglia, we first looked at whether inflammation could regulate Na_v_1.6 expression [49]. Although we detected Na_v_1.6 by immunocytochemistry and saw a positive correlation with inflammation at the level of the *Scn8a* transcript, upon further analysis, we showed that microglia do not express the full length *Scn8a* transcript. Therefore, the observed role of Na_v_1.6 in neuroinflammation may either be an indirect effect on microglial activation or due to other functions that these isoforms may have that are not yet understood [50]. 

We confirmed that tetrodotoxin, a blocker of VGSCs led to a decrease in microglial activation, suggesting that there may be other VGSCs on microglia. Upon further analysis of VGSCs expression in microglia, we showed that Na_v_1.2 is expressed in microglia in vivo during development. Abnormal microglia function during development was shown in *Scn2a* deficient mice in addition to an activated microglial morphology in the adults [51]. To assess the contribution of developmental Na_v_1.2 expression in microglia to this phenotype, a microglia-specific conditional knockout of *Scn2a* would be required. In vitro, the expression of *Scn2a* was modulated by neuroinflammation. In contrast to tetrodotoxin, when we used toxins at the concentrations where previous studies had shown that it blocked Na_v_1.2 currents, [37, 42] we surprisingly saw an increase in inflammation. Interestingly, this agrees with the observation that in Na_v_1.2 knockout animals, compared to control animals, the microglia seem to be more activated [51]. The significance of the expression of VGSCs in microglia is not well understood. Although our results suggest that Na_v_1.2 supresses microglial inflammation, how Na_v_1.2 regulates this remains to be investigated. Elucidating the role of Na_v_1.2 in microglia may be important since it has been observed that not only gain-of-function but also loss-of-function of *Scn2a* results in disease pathology [52].

In conclusion, this study highlights the importance of studying the direct effect of ASMs on microglia, since the effect on microglial activation could play an important role in addressing new ways of overcoming DRE. In addition, this would also, in the future, allow one to assess the status of on-going inflammation in DRE patients and predict which drug would have the most effect.

## Supplementary Information

Below is the link to the electronic supplementary material.

Supplementary file1 (XLSX 26 kb)

Supplementary file2 (DOCX 23753 kb)

## Data Availability

No datasets were generated or analysed during the current study.

## References

[1] Brodie MJ, Shorvon SD, Canger R, Halász P, Johannessen S, Thompson P, et al. Commission on European Affairs: appropriate standards of epilepsy care across Europe. ILEA Epilepsia. 1997;38:1245–50. 9579928 10.1111/j.1528-1157.1997.tb01224.x

[2] Chen Z, Brodie MJ, Liew D, Kwan P. Treatment outcomes in patients with newly diagnosed epilepsy treated with established and new antiepileptic drugs: a 30-year longitudinal cohort study. JAMA Neurol. 2018;75:279–86. 29279892 10.1001/jamaneurol.2017.3949 PMC5885858

[3] Victor TR, Hage Z, Tsirka SE. Prophylactic administration of cannabidiol reduces microglial inflammatory response to kainate-induced seizures and neurogenesis. Neuroscience. 2022;500:1–11. 35700815 10.1016/j.neuroscience.2022.06.010

[4] Sills GJ, Rogawski MA. Mechanisms of action of currently used antiseizure drugs. Neuropharmacology. 2020;168:107966. 32120063 10.1016/j.neuropharm.2020.107966

[5] Villasana-Salazar B, Vezzani A. Neuroinflammation microenvironment sharpens seizure circuit. Neurobiol Dis. 2023;178:106027. 36736598 10.1016/j.nbd.2023.106027

[6] Prabowo AS, Anink JJ, Lammens M, Nellist M, van den Ouweland AM, Adle-Biassette H, et al. Fetal brain lesions in tuberous sclerosis complex: TORC1 activation and inflammation. Brain pathology (Zurich, Switzerland). 2013;23:45–59. 22805177 10.1111/j.1750-3639.2012.00616.x PMC3518755

[7] Vezzani A. Anti-inflammatory drugs in epilepsy: does it impact epileptogenesis? Expert Opin Drug Saf. 2015;14:583–92. 25645535 10.1517/14740338.2015.1010508

[8] Falsaperla R, Collotta AD, Marino SD, Sortino V, Leonardi R, Privitera GF, et al. Drug resistant epilepsies: a multicentre case series of steroid therapy. Seizure Eur J Epilepsy. 2024;117:115–25. 10.1016/j.seizure.2024.02.007 38394725

[9] Grosso S, Farnetani M, Mostardini R, Cordelli D, Berardi R, Balestri P. A comparative study of hydrocortisone versus deflazacort in drug-resistant epilepsy of childhood. Epilepsy Res. 2008;81:80–5. 18524542 10.1016/j.eplepsyres.2008.04.016

[10] Marchi N, Granata T, Freri E, Ciusani E, Ragona F, Puvenna V, et al. Efficacy of anti-inflammatory therapy in a model of acute seizures and in a population of pediatric drug resistant epileptics. PLoS ONE. 2011;6:e18200. 21464890 10.1371/journal.pone.0018200 PMC3065475

[11] Rangarajan A, Mundlamuri RC, Kenchaiah R, Prathyusha PV, Viswanathan LG, Asranna A, et al. Efficacy of pulse intravenous methylprednisolone in epileptic encephalopathy: a randomised controlled trial. J Neurol Neurosurg Psychiatry. 2022;93:1299–305. 36376023 10.1136/jnnp-2022-329027

[12] Eyo UB, Peng J, Swiatkowski P, Mukherjee A, Bispo A, Wu LJ. Neuronal hyperactivity recruits microglial processes via neuronal NMDA receptors and microglial P2Y12 receptors after status epilepticus. J Neurosci Official J Soc Neurosci. 2014;34:10528–40. 10.1523/JNEUROSCI.0416-14.2014 PMC4200107 25100587

[13] Vezzani A, Viviani B. Neuromodulatory properties of inflammatory cytokines and their impact on neuronal excitability. Neuropharmacology. 2015;96:70–82. 25445483 10.1016/j.neuropharm.2014.10.027

[14] Choi J, Nordli DR Jr, Alden TD, DiPatri A Jr, Laux L, Kelley K, et al. Cellular injury and neuroinflammation in children with chronic intractable epilepsy. J Neuroinflammation. 2009;6:38. 20021679 10.1186/1742-2094-6-38 PMC2811703

[15] Okuneva O, Körber I, Li Z, Tian L, Joensuu T, Kopra O, et al. Abnormal microglial activation in the Cstb−/− mouse, a model for progressive myoclonus epilepsy, EPM1. Glia. 2015;63:400–11. 25327891 10.1002/glia.22760

[16] Pascual O, Ben Achour S, Rostaing P, Triller A, Bessis A. Microglia activation triggers astrocyte-mediated modulation of excitatory neurotransmission. Proc Natl Acad Sci U S A. 2012;109:E197-205. 22167804 10.1073/pnas.1111098109 PMC3268269

[17] Dos-Santos-Pereira M, Guimarães FS, Del-Bel E, Raisman-Vozari R, Michel PP. Cannabidiol prevents LPS-induced microglial inflammation by inhibiting ROS/NF-κB-dependent signaling and glucose consumption. Glia. 2020;68:561–73. 31647138 10.1002/glia.23738

[18] Wechsler RT, Burdette DE, Gidal BE, Hyslop A, McGoldrick PE, Thiele EA, et al. Consensus panel recommendations for the optimization of EPIDIOLEX® treatment for seizures associated with Lennox-Gastaut syndrome, Dravet syndrome, and tuberous sclerosis complex. Epilepsia Open. 2024;9:1632–42. 39007525 10.1002/epi4.12956 PMC11450617

[19] Wirrell EC, Lagae L, Scheffer IE, Cross JH, Specchio N, Strzelczyk A. Practical considerations for the use of fenfluramine to manage patients with Dravet syndrome or Lennox-Gastaut syndrome in clinical practice. Epilepsia Open. 2024;9:1643–57. 38962968 10.1002/epi4.12998 PMC11450599

[20] Cha J, Filatov G, Smith SJ, Gammaitoni AR, Lothe A, Reeder T. Fenfluramine increases survival and reduces markers of neurodegeneration in a mouse model of Dravet syndrome. Epilepsia Open. 2024;9:300–13. 38018342 10.1002/epi4.12873 PMC10839300

[21] Wirrell EC, Hood V, Knupp KG, Meskis MA, Nabbout R, Scheffer IE, et al. International consensus on diagnosis and management of Dravet syndrome. Epilepsia. 2022;63:1761–77. 35490361 10.1111/epi.17274 PMC9543220

[22] Craner MJ, Damarjian TG, Liu S, Hains BC, Lo AC, Black JA, et al. Sodium channels contribute to microglia/macrophage activation and function in EAE and MS. Glia. 2005;49:220–9. 15390090 10.1002/glia.20112

[23] Boerma RS, Braun KP, van de Broek MPH, van Berkestijn FMC, Swinkels ME, Hagebeuk EO, et al. Remarkable phenytoin sensitivity in 4 children with SCN8A-related epilepsy: a molecular neuropharmacological approach. Neurotherapeutics. 2016;13:192–7. 26252990 10.1007/s13311-015-0372-8 PMC4720675

[24] Makridis KL, Friedo AL, Kellinghaus C, Losch FP, Schmitz B, Boßelmann C, Kaindl AM. Successful treatment of adult Dravet syndrome patients with cenobamate. Epilepsia. 2022;63(12):e164–71. 36176237 10.1111/epi.17427

[25] Anderson LL, Hawkins NA, Thompson CH, Kearney JA, George AL Jr. Unexpected efficacy of a novel sodium channel modulator in Dravet syndrome. Sci Rep. 2017;7:1682. 28490751 10.1038/s41598-017-01851-9 PMC5431801

[26] Zelenka L, Pägelow D, Krüger C, Seele J, Ebner F, Rausch S, et al. Novel protocol for the isolation of highly purified neonatal murine microglia and astrocytes. J Neurosci Methods. 2022;366:109420. 34808220 10.1016/j.jneumeth.2021.109420

[27] Boccazzi M, Van Steenwinckel J, Schang AL, Faivre V, Le Charpentier T, Bokobza C, et al. The immune-inflammatory response of oligodendrocytes in a murine model of preterm white matter injury: the role of TLR3 activation. Cell Death Dis. 2021;12:166. 33558485 10.1038/s41419-021-03446-9 PMC7870670

[28] Bokobza C, Joshi P, Schang AL, Csaba Z, Faivre V, Montané A, et al. MiR-146b protects the perinatal brain against microglia-induced hypomyelination. Ann Neurol. 2022;91:48–65. 34741343 10.1002/ana.26263 PMC9298799

[29] Livak KJ, Schmittgen TD. Analysis of relative gene expression data using real-time quantitative PCR and the 2(-Delta Delta C(T)) method. Methods. 2001;25:402–8. 11846609 10.1006/meth.2001.1262

[30] Vezzani A, Balosso S, Ravizza T. Neuroinflammatory pathways as treatment targets and biomarkers in epilepsy. Nat Rev Neurol. 2019;15:459–72. 31263255 10.1038/s41582-019-0217-x

[31] Zographos GA, Russ-Hall SJ, Scheffer IE. Does long-term phenytoin have a place in Dravet syndrome? Ann Clin Transl Neurol. 2022;9:2036–40. 36314457 10.1002/acn3.51684 PMC9735367

[32] Paolicelli RC, Sierra A, Stevens B, Tremblay M-E, Aguzzi A, Ajami B, et al. Microglia states and nomenclature: a field at its crossroads. Neuron. 2022;110:3458–83. 36327895 10.1016/j.neuron.2022.10.020 PMC9999291

[33] Pinto MV, Fernandes A. Microglial phagocytosis-rational but challenging therapeutic target in multiple sclerosis. Int J Mol Sci. 2020. 10.3390/ijms21175960. 32825077 10.3390/ijms21175960 PMC7504120

[34] Guerrini R, Conti V, Mantegazza M, Balestrini S, Galanopoulou AS, Benfenati F. Developmental and epileptic encephalopathies: from genetic heterogeneity to phenotypic continuum. Physiol Rev. 2023;103:433–513. 35951482 10.1152/physrev.00063.2021 PMC9576177

[35] Black JA, Liu S, Waxman SG. Sodium channel activity modulates multiple functions in microglia. Glia. 2009;57:1072–81. 19115387 10.1002/glia.20830

[36] Hossain MM, Sonsalla PK, Richardson JR. Coordinated role of voltage-gated sodium channels and the Na+/H+ exchanger in sustaining microglial activation during inflammation. Toxicol Appl Pharmacol. 2013;273:355–64. 24070585 10.1016/j.taap.2013.09.011 PMC3874798

[37] Almog Y, Fadila S, Brusel M, Mavashov A, Anderson K, Rubinstein M. Developmental alterations in firing properties of hippocampal CA1 inhibitory and excitatory neurons in a mouse model of Dravet syndrome. Neurobiol Dis. 2021;148:105209. 33271326 10.1016/j.nbd.2020.105209

[38] Hossain MM, Weig B, Reuhl K, Gearing M, Wu LJ, Richardson JR. The anti-parkinsonian drug zonisamide reduces neuroinflammation: role of microglial Na(v) 1.6. Exp Neurol. 2018;308:111–9. 30017881 10.1016/j.expneurol.2018.07.005 PMC7404626

[39] O’Brien JE, Drews VL, Jones JM, Dugas JC, Barres BA, Meisler MH. Rbfox proteins regulate alternative splicing of neuronal sodium channel SCN8A. Mol Cell Neurosci. 2012;49:120–6. 22044765 10.1016/j.mcn.2011.10.005 PMC3278527

[40] Plummer NW, McBurney MW, Meisler MH. Alternative splicing of the sodium channel SCN8A predicts a truncated two-domain protein in fetal brain and non-neuronal cells*. J Biol Chem. 1997;272:24008–15. 9295353 10.1074/jbc.272.38.24008

[41] Han Z, Chen C, Christiansen A, Ji S, Lin Q, Anumonwo C, et al. Antisense oligonucleotides increase Scn1a expression and reduce seizures and SUDEP incidence in a mouse model of Dravet syndrome. Sci Transl Med. 2020;12:eaaz6100. 32848094 10.1126/scitranslmed.aaz6100

[42] Gould E, Kim JH. *SCN2A* contributes to oligodendroglia excitability and development in the mammalian brain. Cell Rep. 2021;36:109653. 34496232 10.1016/j.celrep.2021.109653 PMC8486143

[43] Rawat C, Kukal S, Dahiya UR, Kukreti R. Cyclooxygenase-2 (COX-2) inhibitors: future therapeutic strategies for epilepsy management. J Neuroinflammation. 2019;16:197. 31666079 10.1186/s12974-019-1592-3 PMC6822425

[44] Fitzpatrick J-M, Minogue E, Curham L, Tyrrell H, Gavigan P, Hind W, et al. MyD88-dependent and -independent signalling via TLR3 and TLR4 are differentially modulated by Δ9-tetrahydrocannabinol and cannabidiol in human macrophages. J Neuroimmunol. 2020;343:577217. 32244040 10.1016/j.jneuroim.2020.577217

[45] Di Sapia R, Zimmer TS, Kebede V, Balosso S, Ravizza T, Sorrentino D, et al. CXCL1-CXCR1/2 signaling is induced in human temporal lobe epilepsy and contributes to seizures in a murine model of acquired epilepsy. Neurobiol Dis. 2021;158:105468. 34358616 10.1016/j.nbd.2021.105468

[46] Nakamura M, Cho J-H, Shin H, Jang I-S. Effects of cenobamate (YKP3089), a newly developed anti-epileptic drug, on voltage-gated sodium channels in rat hippocampal CA3 neurons. Eur J Pharmacol. 2019;855:175–82. 31063770 10.1016/j.ejphar.2019.05.007

[47] Broekaart DWM, Anink JJ, Baayen JC, Idema S, de Vries HE, Aronica E, et al. Activation of the innate immune system is evident throughout epileptogenesis and is associated with blood-brain barrier dysfunction and seizure progression. Epilepsia. 2018;59:1931–44. 30194729 10.1111/epi.14550

[48] de Vries EE, van den Munckhof B, Braun KP, van Royen-Kerkhof A, de Jager W, Jansen FE. Inflammatory mediators in human epilepsy: a systematic review and meta-analysis. Neurosci Biobehav Rev. 2016;63:177–90. 26877106 10.1016/j.neubiorev.2016.02.007

[49] Li X, Wu X, Li N, Li D, Sui A, Khan K, et al. Scorpion venom heat-resistant synthesized peptide ameliorates 6-OHDA-induced neurotoxicity and neuroinflammation: likely role of Nav1.6 inhibition in microglia. Br J Pharmacol. 2021;178:3553–69. 33886140 10.1111/bph.15502

[50] Alrashdi B, Dawod B, Tacke S, Kuerten S, Côté PD, Marshall JS. Mice heterozygous for the sodium channel Scn8a (Nav1.6) have reduced inflammatory responses during EAE and following LPS challenge. Front Immunol. 2021. 10.3389/fimmu.2021.533423. 33815353 10.3389/fimmu.2021.533423 PMC8017164

[51] Wu J, Zhang J, Chen X, Wettschurack K, Que Z, Deming BA, et al. Microglial over-pruning of synapses during development in autism-associated SCN2A-deficient mice and human cerebral organoids. Mol Psychiatry. 2024. 10.1038/s41380-024-02518-4. 38499656 10.1038/s41380-024-02518-4

[52] Spratt PWE, Alexander RPD, Ben-Shalom R, Sahagun A, Kyoung H, Keeshen CM, et al. Paradoxical hyperexcitability from Na(V)1.2 sodium channel loss in neocortical pyramidal cells. Cell Rep. 2021;36:109483. 34348157 10.1016/j.celrep.2021.109483 PMC8719649

