# Supplementary material for: Microglial activation is inhibited by selective anti-seizure medications
Source: Inflamm Res. 2025 Aug 23;74(1):112. doi: 10.1007/s00011-025-02076-7 (PMC12374903; doi:10.1007/s00011-025-02076-7)
Supplement: Supplementary file 2 — Supplementary file2 (DOCX 23753 kb) [file 11_2025_2076_MOESM2_ESM.docx]

**Supplementary Information**

**Materials and Methods**

Preparation of microglial cultures: Mouse pups were sacrificed by decapitation, the brains were dissected in Hanks Balaced Salt Solution (HBSS) so that only the cortices remained, the remaining components were discarded. The meninges of the cortices were removed, the cortices were minced with a scalpel and stored on ice in a 15 mL Falcon tube in HBSS (max. 15 brains per 15 mL Falcon). HBSS was removed and 3 mL of 2.5% trypsin was added to the cortices, the cortices were incubated in the trypsin for 25 min at 37 ℃. After incubation, the trypsin was neutralized with 5 mL fetal bovine serum (FBS) and the mixture was transferred to a 50 mL Falcon tube. The cortices were further disrupted by pipetting up and down 10 times with a 5 mL pipette, then 100 μL DNAse I was added. The tube was incubated for 1 min at room temperature, then the cortices are centrifuged down (270 rcf, 5 min, 4 ℃), and the supernatant was removed. The pellet was resuspended in DMEM (Dulbecco's Modified Eagle Medium), containing 10% fetal bovine serum and 1% penicillin-streptomycin and filtered through a 70 μm cell filter. The DMEM with the cells was then transferred to T75 cell bottles (7 bottles for 15 brains) and these were filled up to 12 mL with DMEM. Approximately 24 hours later, the DMEM in the bottles was exchanged with fresh DMEM medium. At 7 days after dissection, 5 mL of DMEM was added to each bottle.

Twelve days after dissection, the microglia were transferred to well plates. To do so, the bottles were shaken for 25 minutes at 250 rpm, the DMEM was transferred to 50 ml Falcon tubes and centrifuged at 280 rcf for 5 minutes at 4 ℃. The supernatant was removed and the pellet resuspended in DMEM. Cells were counted and seeded into well plates at the required number (for qPCR: 150.000 cells per well of a 24-well plate, for immunocytochemistry 30.000 per coverslip). After 24 hours, the DMEM was replaced with Macrophage-SFM (serum free medium) containing 1% penicillin-streptomycin. The cells were stimulated 2-4 days after seeding.

**Scheme of stimulation paradigm**


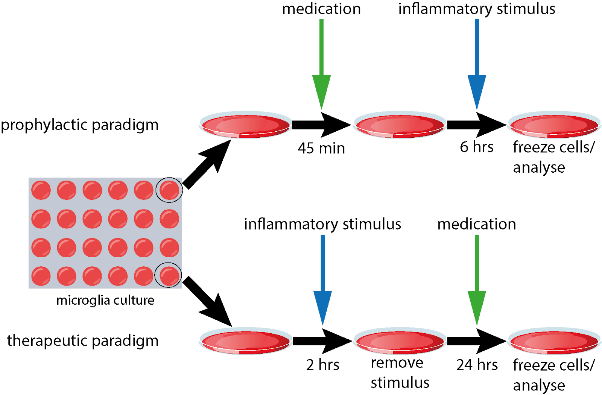


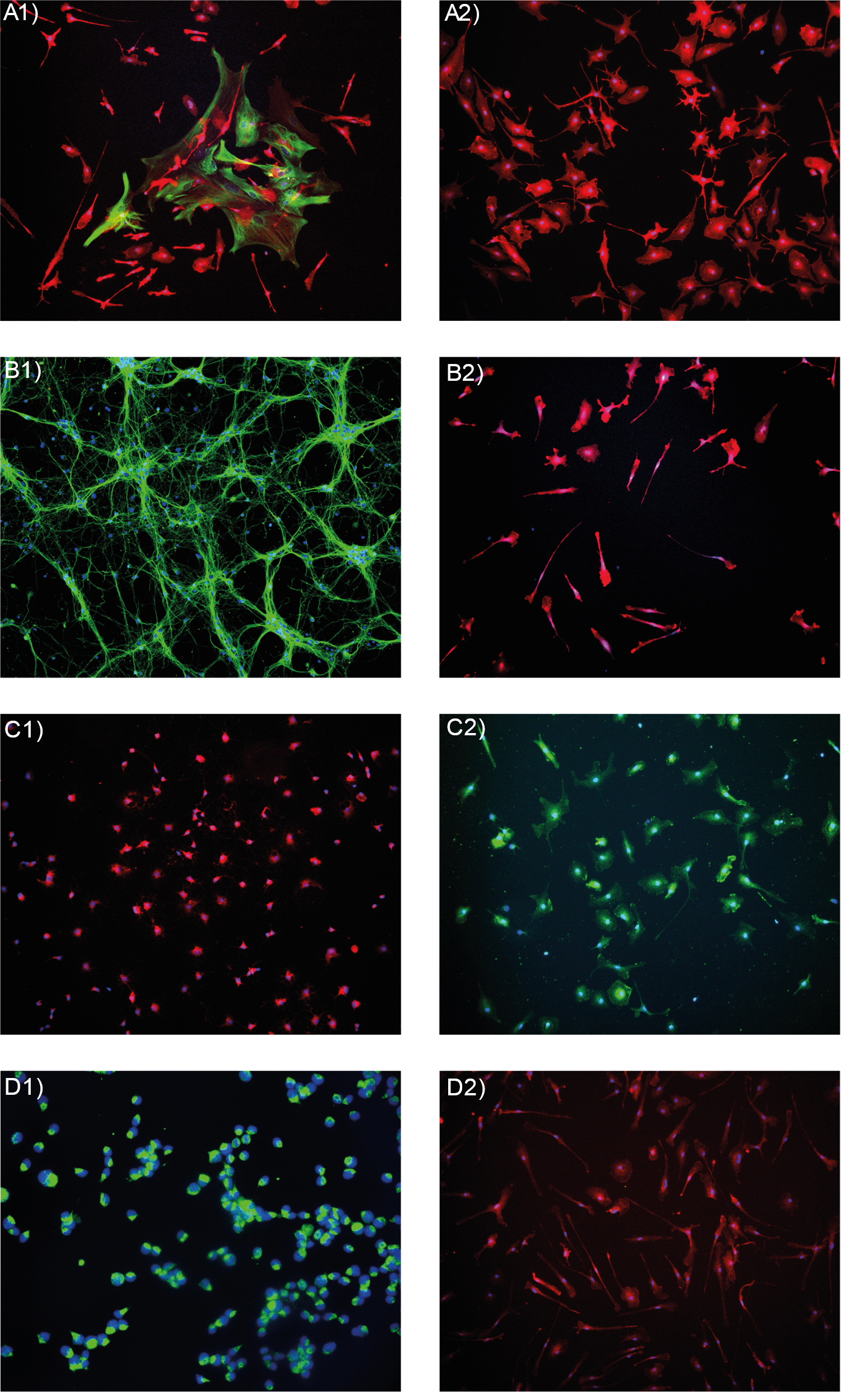


**Supplementary figure 1: Contamination Staining.** Contamination staining for astrocytes (GFAP) A1 and A2, Neurons (TUJ1) B1 and B2, oligodendrocytes (MBP) C1 and C2 and oligodendrocyte precursor cells (NG2) D1 and D2. Shown are a positive control (B1, C1, D1) and the corresponding microglia staining (A2, B2, C2, D2), A1 shows a rare example of astrocytes, A2 shows representative microglia culture featuring a majority of cells staining positive for Iba1. A1 and 2: Iba1 (red), GFAP (green), DAPI (blue); B1 and 2: Iba1 (red), TUJ1 (green), DAPI (blue); C1 and 2: MBP (red), Iba1 (green), DAPI blue; D1 and 2: Iba1 (red), NG2 (green), DAPI (blue).

**Supplementary Figures**


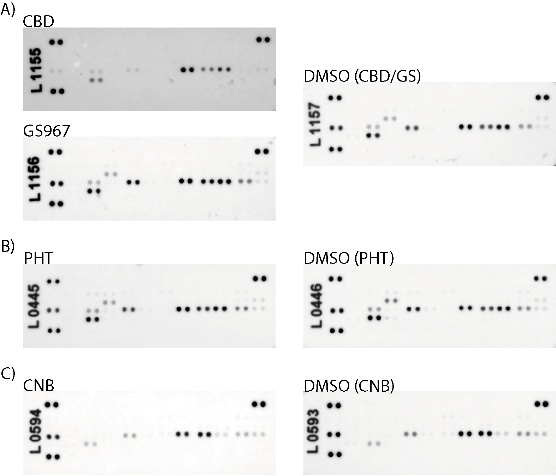


**Supplementary figure 2: Associated membranes for figure 3 graphs (shown are the membranes from the first “n”)**


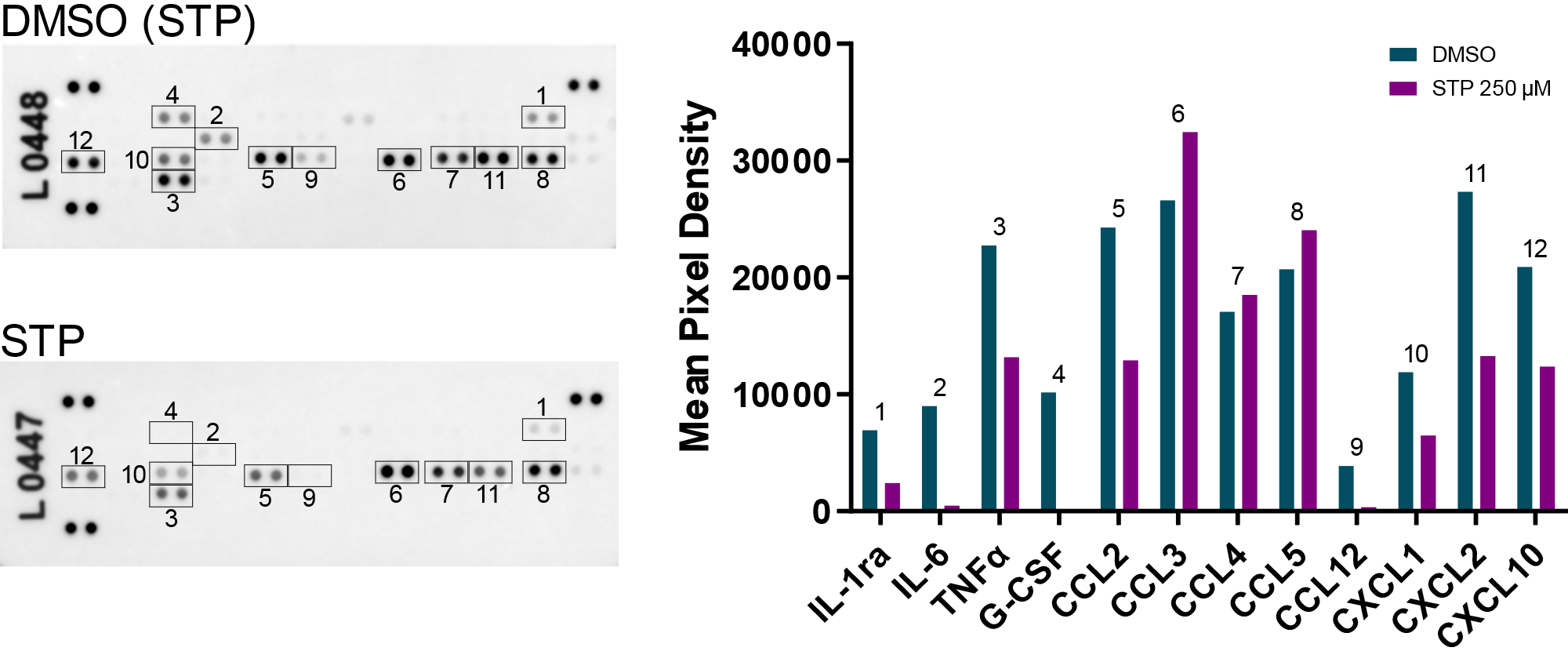


**Supplementary figure 3:** Effect of STP on the release of selected cyto- and chemokines on microglial cells pre-stimulated with LPS (10 μg/mL) for two hours followed with only STP for 24h. (n=1) Abbreviations: DMSO = dimethyl sulfoxide, STP = stiripentol

**
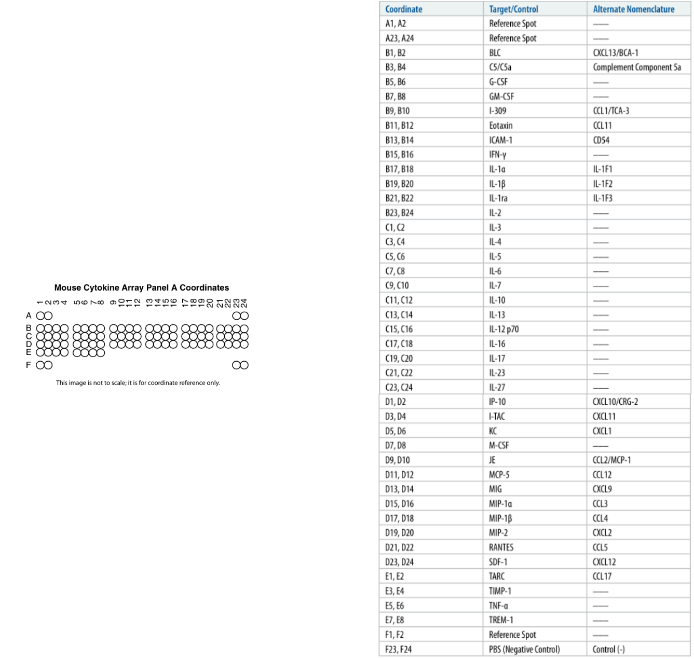
Supplementary figure 4: Reference material from R&D systems for analysing cytokine membranes.**

| paradigm | effect on | CBD 10 µM | FFA 50 µM | STP 250 µM | PHT 8 µM | PHT 40 µM | CNB 900 µM | GS967 10 µM | GS967 30 µM |
| --- | --- | --- | --- | --- | --- | --- | --- | --- | --- |
| prophylactic | *Ptgs2* | ↓* | ns | ns | ↓** | ↓* | ns | ns | ↓** |
|  | *Tnf-α* | ↓* | ns | ns | ns | ns | ns | ↓* | ns |
|  | *Ifn-β* | ns | ns | ns | ↑** | ns | ↓* | ns | ns |
| therapeutic | *Ptgs2* | ↓** | ↑* | ↓** | ns | ns | - | - | ↓* |
|  | *Ifn-β* | - | - | - | ns | ns | ns | - | - |
|  | area | ↓**** | ns | ↓**** | ns | ns | ns | - | ns |
|  | circularity | ↓**** | ns | ↓**** | ns | ns | ↓*** | - | ↓**** |

**Supplementary figure 5: Summary of results.**

*p< 0.05, **p< 0.01, ***p< 0.001, ****p< 0.0001 Abbreviations: CBD = cannabidiol, CNB = cenobamate, FFA = fenfluramine, PHT = Phenytoin, STP = stiripentol, ns = not significant
